# India: Intruder Node Detection and Isolation Action in Mobile Ad Hoc Networks Using Feature Optimization and Classification Approach

**DOI:** 10.1007/s10916-019-1309-2

**Published:** 2019-05-10

**Authors:** T. Kavitha, K. Geetha, R. Muthaiah

**Affiliations:** School of Computing, SASTRA Deemed to be University, Thanjavur, India

**Keywords:** MANET, Intrusion detection system, Malicious node detection, Feature extraction, Feature optimization, Classification

## Abstract

Due to lack of a central bureaucrat in mobile ad hoc networks, the security of the network becomes serious issue. During malicious attacks, according to the motivation of intruder the severity of the threat varies. It may lead to loss of data, energy or throughput. This paper proposes a lightweight Intruder Node Detection and Isolation Action mechanism (INDIA) using feature extraction, feature optimization and classification techniques. The indirect and direct trust features are extracted from each node and the total trust feature is computed by combining them. The trust features are extracted from each node of MANET and these features are optimized using Particle Swarm Optimization (PSO) algorithm as feature optimization technique. These optimized feature sets are then classified using Neural Networks (NN) classifier which identifies the intruder node. The performance of the proposed methodology is studied in terms of various parameters such as success rate in packet delivery, delay in communication and the amount of energy consumption for identifying and isolating the intruder.

## Introduction

**Fig. 1:**
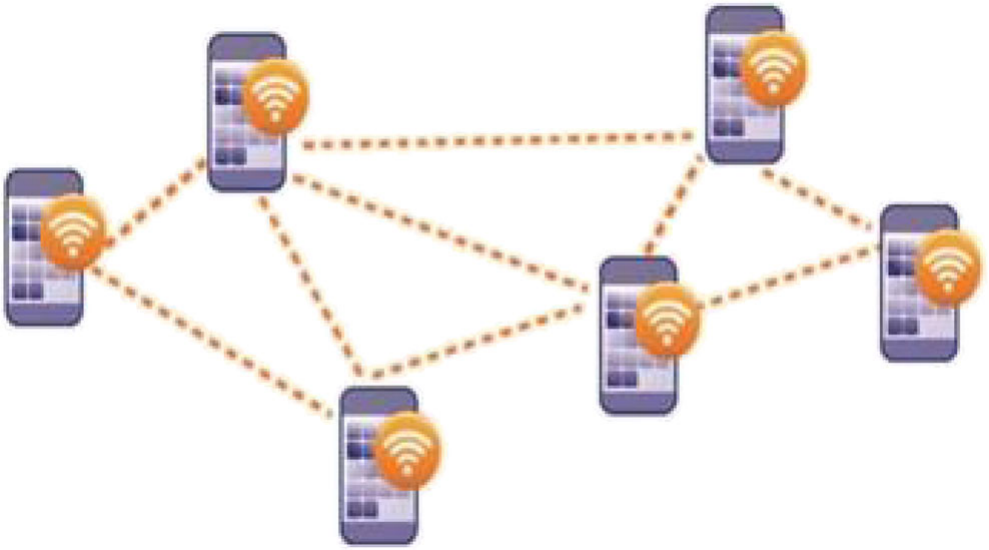
MANET architecture

Wireless communication technology play a vital role in transient communication nowadays. Numerous end users are linked by wireless technology through diversified wireless devices. The cost and size of wireless devices are significantly reduced over the past years. MANET is one of the wireless network paradigm to support high volume of end users. In MANET, the mobile devices are allowed to move in any direction within the allocated range or area coverage. The major applications of MANET are military environment, disaster recovery and conventional road traffic. Figure 1 shows the architecture of MANET in which numbers of mobile nodes are connected with each other in wireless mode.

Many research works dealt to improve the performance of MANET in terms of security [1–3]. The security of individual nodes is very important in MANET in order to protect the private data of individual user. An intruder may provide false route reply messages to get the attention of senders for attracting the data packets and then junks / misuse the data [4]. This paper proposes a methodology to sense the malicious nodes in MANET using feature optimization technique incorporated with classification approaches.

The conventional practices for malicious node detection are reviewed in section 2 and section 3 elaborates the proposed methodology based on feature optimization and classification approach. Section 4 discusses the simulation results of this paper and section 5 concludes the paper.

## Literature survey

The authors of [5] used clustering algorithm for spotting and mitigating the intruders in MANET. The authors grouped the nodes of a MANET using clustering concept and the clustered groups are analyzed further to sense the malicious nodes. In [6], they utilized Watchdog protocol to detect and classify the malicious nodes in Destination-Sequenced Distance Vector routing (DSDV) routing methodology. The proposed wok detected and classified link failures due to the availability of malicious nodes. The authors of [7] used cooperative bait detection scheme for malicious node detection in MANET environment. The authors detected and classified grayhole or collaborative blackhole attacks in MANET system. The routing in MANET was carried out using best-effort fault-tolerant routing methodology and the authors analyzed the performance of the projected system in terms of packet delivery ratio and routing overhead. In [8], the proposed method uses a heterogeneous algorithm for malicious node detection in MANET. This algorithm was based on constructing the links between trusty and untrusted nodes in network environment. Periodical dropping of packets were detected and the performance was carried out based on dropped packets ratio. The work proposed in [9] used route establishment technique and packets forwarding algorithm for spotting and mitigating the malevolent nodes in MANET. The researchers achieved 80% of the detection rate for their proposed algorithm for malicious node detection. A hybrid defense algorithm is proposed in [10] for the detection of malicious nodes in MANET environment. The authors used bait detection scheme to improve the quality factor of the system. Many researches have proposed solutions to mitigate single node attacks [11–13], but they prevent the network from collaborative attacks when the malicious node is not compromised with other non-legitimate nodes. But they cannot protect the network from attacks such as wormhole attack. The fuzzy based approaches [14, 15] may not be a better solution for network lifetime improvement, as the energy of individual nodes are not taken into account.

## INDIA: Intruder node detection and isolation action

**Fig. 2:**
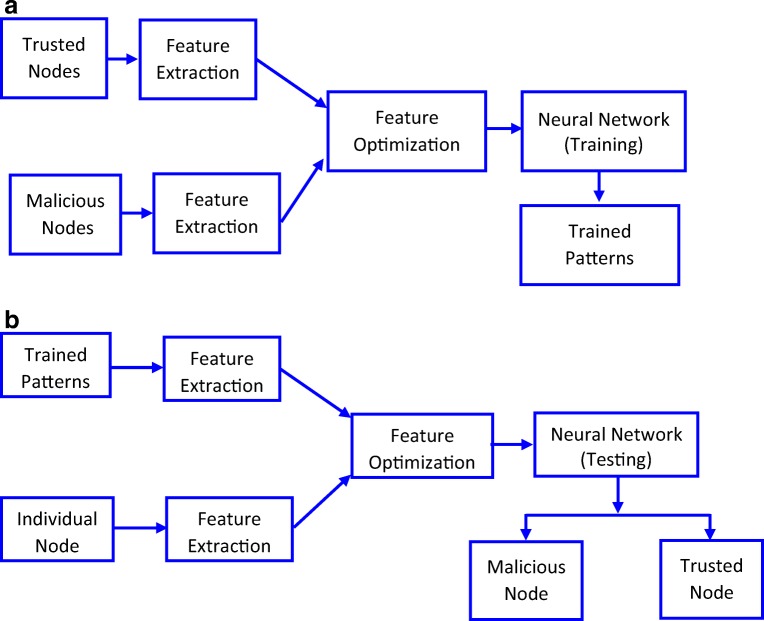
**a** Training Module of INDIA System. **b** Testing Module of INDIA System

The working stream of INDIA mechanism is illustrated in Fig. 2a and b. Figure 2a shows the training module of INDIA mechanism. In training mode of the system, the features from both trusted nodes and malicious nodes of a well-known MANET are extracted. These features are optimized using PSO algorithm in order to improve the classification accuracy to maximum level for malicious node detection process.

The testing mode of the proposed system is depicted in Fig. 2b. In testing mode of the scheme, the features are extracted from each node and these features are classified based on the trained patterns.

### Feature extraction

**Fig. 3:**
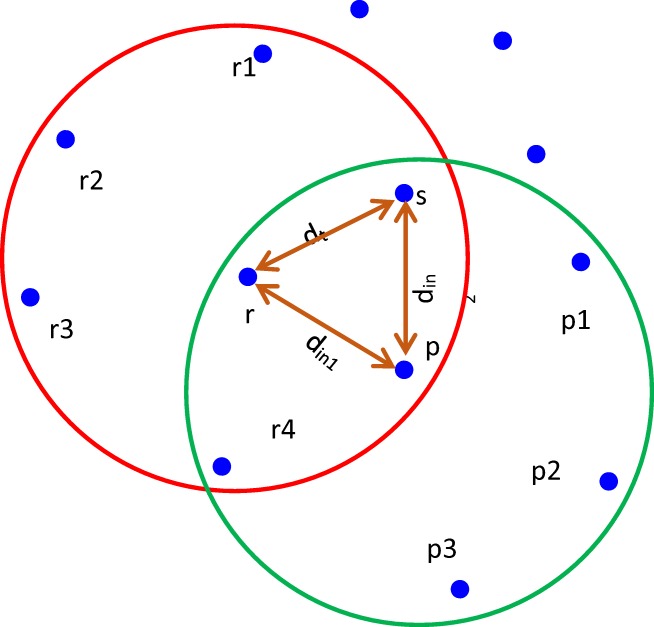
Trust value estimation on r by s

**Fig. 4:**
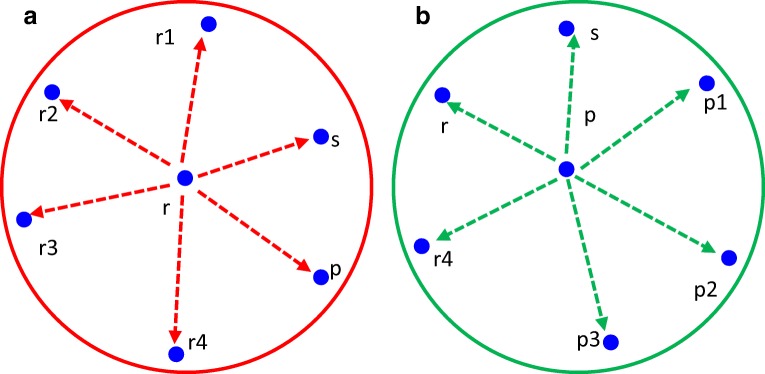
**a** Trust value estimation on p by r. **b** Trust value estimation on r by s through p

Figure 3 shows the trust value estimation on r by s. Figure 4a shows the trust value estimation on p by r and Fig. 4b describes the trust value estimation on r by s through p. The direct and indirect features are extracted and their individual trust values are estimated. If the features are extracted from node ‘r’, then the surrounding nodes over the node ‘r’ is r1,r2,r3,r4,p and s.

The direct trust value is calculated as,

$$ {d}_t=\sum \limits_{i=1}^{N_1}{\left(i-\mu \right)}^2\times {P}_i $$

where, the probability metric is represented by P_i_, the average number of packets acquired by r over the time period ‘t’. The average number of packets acquired by node ‘r’ over the time period ‘t’.

The probability metric of each individual node is calculated as,

$$ {P}_i=\frac{\alpha_i-{\beta}_i}{\alpha_i} $$

where, *α*_*i*_ is the number of packets retrieved over time ‘t’ and *β*_*i*_ is the number of packets conveyed over the time ‘t’.

The trust estimation between nodes ‘r’ and ‘p’ is,

$$ {d}_{in1}=\sum \limits_{i=1}^{N_1}{\left(i-\mu \right)}^2\times {P}_i\times {W}_i $$

where, N_1_ is the sum of neighboring nodes over the node p.

The weight of individual node with respect to node p can be computed as,

$$ {w}_i=\frac{\sum \limits_{i=1}^N\ {P}_i\times {X}_i}{k} $$

where, k is kappa factor and it is given as,

$$ k=\sum {P}_i $$

The trust estimation between nodes ‘p’ and ‘s’ is,

$$ {d}_{in2}=\sum \limits_{i=1}^{N_2}{\left(i-\mu \right)}^2\times {P}_i\times {W}_i $$

where, N_2_ is the number of neighboring nodes over the node s.

The total indirect trust is given as,

$$ {d}_{in}={d}_{in1}+{d}_{in2} $$

Hence, total trust of the individual node ‘r’ is given as,

$$ Total\ Trust={d}_d+{d}_{in} $$

### Feature optimization

The extracted features are then optimized using PSO algorithm in order to improve the malicious node detection rate. This PSO algorithm based feature optimization works as follows:

- *Step 1:* Determine the population size, speed and position or coordinates of each particles; Initialize all these parameters for optimization.
- *Step 2:* The population of the particles can be generated by,

$$ {X}_i={\left\{{x}_1,{x}_2,{x}_3\dots \dots {x}_N\right\}}^T $$

where, N is the total number of particles in population vector or list and x is the particles.

- *Step 3:* Determine the fitness value of every particle in population vector using the following equations as,

$$ {f}_i=\sum \limits_{k=1}^{N-1}{\left({x}_k-\overline{x_k}\right)}^2 $$

where, N is the number of particles in population vector or list and $\overline{x_k}$ is the mean of the population.

- *Step 4:* Update the optimal fitness value of each population as Pbest.
- *Step 5:* Update the population fitness value of each population as Gbest.
- *Step 6:* The optimization metric can be estimated using the following equation as,

$$ OPT\_M=-\sum \limits_{k=1}^N{\left(\frac{f_i-{f}_a}{f_i}\right)}^2 $$

where, *f*_*a*_ is the average value of the fitness values.

- *Step 7:* Keep informed the current position and speed of each particles in population list and follow the steps from 1 to 5.

### Classifications

**Fig. 5:**
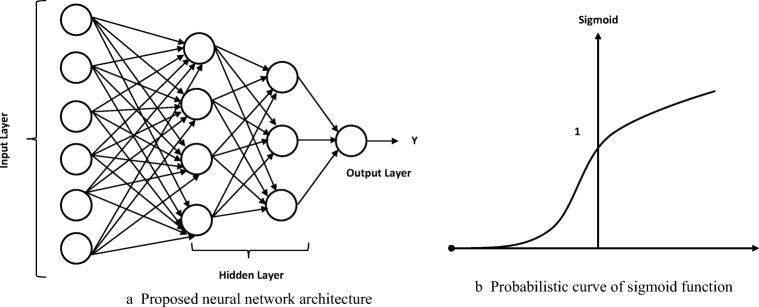
**a** Proposed neural network architecture. **b** Probabilistic curve of sigmoid function

The linear mapping of input and output samples is achieved using back propagation neural network classification approach. The error rate of this classification approach is significantly reduced due to its weight and the threshold values of the intermediate layers in neural networks. In the proposed methodology, two hidden layer incorporated with single output layer is designed to achieve high classification rate and to reduce the error rate. In order to improve the classification rate of the classification approach, sigmoid function is adopted. The proposed neural network can be operated into three modes as training mode, validation mode and testing mode. The extracted features from the set of nodes of a MANET are divided into training, validation and testing features. In case of training mode, the well-known features from both malicious and legitimate nodes are fed into designed neural network to get the trained samples or patterns. Validation mode is utilized to determine whether the training of the MANET is enough to reduce the error rate. After the validation mode is executed, the features from each node in the network are tested using trained and validation patterns to achieve low error rate. The proposed neural network architecture constitutes six nodes in input layer, two hidden layer which incorporates 4 and 3 nodes and 1node in output layer. The proposed neural network architecture and its probabilistic curve of sigmoid function are illustrated in Fig. 5a and b respectively.

The sigmoid function of the proposed neural network is given in the following equation as,

$$ f(x)=\frac{1}{1+{e}^{-\beta x}} $$

where, β is the beta function which ranging from 0 and 1. The architecture produces two classes as class low and class high. The class low is set as output response for non-malicious node in MANET and class high is set as output response for malicious node in MANET environment. Each node in MANET environment is classified as malicious or legitimate node using the classification approach. The identified intruders are then isolated / quarantined by adding them with block list.

## Results and discussions

To accomplish the work NetSim standard version network simulator was used to implement the nodes in MANET and to estimate the performance of INDIA mechanism to detect the malicious nodes. The proposed intruder detection system was trained in both static and dynamic environments. In case of static environment, the nodes in MANET are fixed in its coordinates and in case of dynamic environment the nodes are freely movable from one position to another position. For evaluating the performance of the system, 100 nodes are considered and the in-house PACR [16] is used to send the packets from source to destination.

The performance of INDIA mechanism is analyzed in terms of rate of successful packet delivery, time consumption in communication as well as identifying the intruder for isolation and energy consumption for the complete process. To analyze the performance of the MANET in high level of accuracy, 25 nodes in MANET are set into intruders. The general characteristics of these 25 nodes are altered and the performance of the MANET is analyzed based on these intruders. The packet delivery ratio is defined as the ratio between number of packets properly transferred from source to destination and the total number of packets. Normally, the measured packet delivery ratio may vary from 0% to 100% with respect to the number of compromised nodes in the network.

**Fig. 6:**
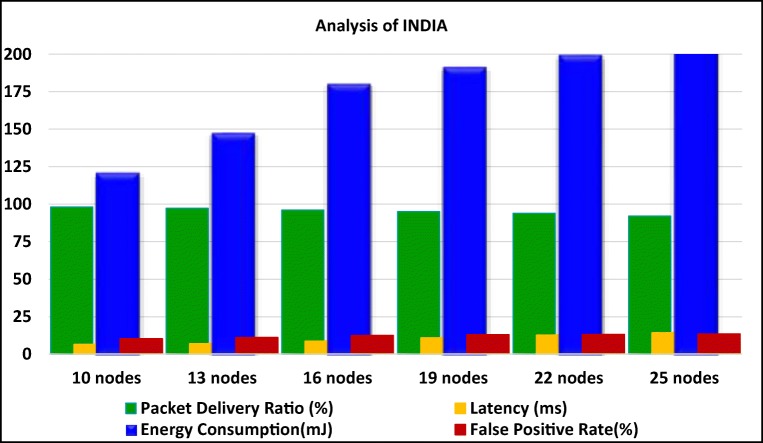
Analysis of PDR, Latency, Energy Consumption and FPR

The measured packet delivery rate shows that the proposed method works better in identifying the intruder and isolating it. Figure 6 shows the packet delivery ratio (PDR) of a MANET with 100 nodes. It also shows the analysis of latency with respect to various numbers of malevolent nodes in MANET environment. Latency is defined as the time utilized for spotting the malicious nodes in MANET. The latency value is high when the number of malicious nodes grows. It took minimal time to detect the intruder though they are high in number. Figure 6 shows the analysis of energy consumption with respect to various numbers of malevolent nodes in MANET. The number of malicious nodes may increases the energy consumption. But as the proposed protocol was able to identify the intruders swiftly, the network lifetime is conserved. Figure 6 analyses the performance of INDIA mechanism in terms of False Positive Rate (FPR). It was apparent from the results that the proposed system do not increase in FPR with respect to the number of intruders.

Table 1 shows the performance comparison of INDIA approach for intruder identification with and without feature optimization. The proposed work proves to be better when feature optimization is added. Table 2 describes the performance comparison of INDIA approach with respect to conventional methodologies in terms of PDR, latency and energy consumption at 10% of malicious nodes presence in the network. The conventional methodologies for malicious node detection used cooperative bait detection algorithm whereas the proposed methodology INDIA used in this paper devised feature optimization based classification approach for improving the performance of the malevolent node discovery in MANET.

**Table 1:** Performance comparison of INDIA with and without feature optimization

Performance evaluation parameters	INDIA	
	Without feature optimization	With feature optimization
PDR (%)	93	98
Latency (ms)	16.7	9.7
Energy consumption (mJ)	179.5	120.5

**Table 2:** Performance comparison of INDIA approach with conventional methodologies

Performance evaluation parameters	Proposed work (with feature optimization)	Bait Detection Approach [7]	Clustering Approach [5]
PDR (%)	98	92	89
Latency (ms)	9.7	12.8	15.9
Energy consumption (mJ)	120.5	154.7	169.5

## Conclusions

In this paper, Intruder Node Identification and Isolation Action mechanism using feature optimization and classification approach is proposed. Particle Swarm Optimization algorithm is used to optimize the extracted direct and indirect trust features. These features are classified using Neural Network classifier. The proposed work without optimization methodology achieves 93% of packet delivery rate, 16.7 ms of latency and 179.5 mJ of energy consumed at 10% of malicious nodes presence in MANET. The proposed work with optimization methodology achieves 98% of PDR, 9.7 ms of latency and 120.5 mJ of energy consumption at 10% of malicious nodes present in a MANET.
